# Frontal lobe changes occur early in the course of affective disorders in young people

**DOI:** 10.1186/1471-244X-12-4

**Published:** 2012-01-20

**Authors:** Jim Lagopoulos, Daniel F Hermens, Sharon L Naismith, Elizabeth M Scott, Ian B Hickie

**Affiliations:** 1 Clinical Research Unit, Brain and Mind Research Institute, University of Sydney, Australia

**Keywords:** VBM, clinical staging, psychosis, bipolar disorder, depression, MRI

## Abstract

**Background:**

More severe and persistent forms of affective disorders are accompanied by grey matter loss in key frontal and temporal structures. It is unclear whether such changes precede the onset of illness, occur early in the course or develop gradually with persistence or recurrence of illness. A total of 47 young people presenting with admixtures of depressive and psychotic symptoms were recruited from specialist early intervention services along with 33 age matched healthy control subjects. All participants underwent magnetic resonance imaging and patients were rated clinically as to current stage of illness. Twenty-three patients were identified as being at an early 'attenuated syndrome' stage, while the remaining were rated as having already reached the 'discrete disorder' or 'persistent or recurrent illness' stage. Contrasts were carried out between controls subjects and patients cohorts with attenuated syndromes and discrete disorders, separately.

**Results:**

The patients that were identified as having a discrete or persisting disorder demonstrated decreased grey matter volumes within distributed frontal brain regions when contrasted to both the control subjects as well as those patients in the attenuated syndrome stage. Overall, patients who were diagnosed as more advanced in terms of the clinical stage of their illness, exhibited the greatest grey matter volume loss of all groups.

**Conclusions:**

This study suggests that, in terms of frontal grey matter changes, a major transition point may occur in the course of affective illness between early attenuated syndromes and later discrete illness stages.

## Background

Structural neuroimaging studies in patients with established affective and psychotic disorders have emphasised the extent to which discrete regions of fronto-temporal grey matter are selectively diminished [1-3]. Most studies, however, have focused on patient cohorts at the more severe and persistent illness end of the spectrum [4]. In some studies, key structural changes such as reduced hippocampal volume have been more marked in late-onset, as distinct from early onset, cases [5-7]. There is a paucity of studies that have characterised structural brain changes prior to onset or during the early stages of these disorders. As compared with studies related to psychosis onset [2,8] little is known about potential grey matter changes that occur when patients transition from earlier to later phases of illness. This is particularly pertinent, since better understanding the trajectory of the disease process may provide enhanced opportunities for the development of more effective early intervention strategies [9-12].

To this end, we have developed a clinical staging model for application in the early phases of illness of patients who present with admixtures of anxiety, depressive or psychotic symptoms [13]. It attempts to identify closer links between early clinical phenotypes and discrete pathways to illness [14,15]. Young help seeking individuals with mixed symptoms are typically closer to their illness onset than middle-aged adults with discrete disorders. "Detailed clinical and neurobiological examination of these individuals (in cross-section and longitudinally) may provide a window of opportunity for identifying core neurobiological characteristics of the illness (i.e. disease biomarkers) that are independent of the effects of persisting or recurrent illness, comorbid alcohol or other substance misuse."

The principal aim of this study was to test whether there are distinct patterns of grey matter changes evident very early in the course of affective illness and whether these patterns differ from those seen in subjects who have progressed to later stages. We hypothesised that there would be a gradation of structural deficits associated with transition from early to later clinical stages. Specifically, we hypothesised that grey matter changes would be more extensive in those with discrete disorders (and as such at later stages of illness), as compared to those with earlier sub-syndromal or attenuated syndromes.

## Results

### Sample characteristics

Whilst the various clinical stages did not differ in terms of the education (years) they did differ in terms of age [t (df = 38.6) = -2.4, p=.023], with the help-seeking/attenuated syndrome (stage 1) group being approximately 3 years younger than the discrete disorder/persistent illness group (stages 2/3). The stage 1 group as compared with the stage 2/3 group did not differ in predicted IQ (see Table 1); nor did they differ in clinical ratings for current social and occupational functioning (SOFAS), depressive symptoms (HDRS), self-reported psychological distress (K10), or general psychiatric symptoms (indexed by BPRS total; see Table 1).

**Table 1:** Demographic and clinical variables

	Healthy Controls [N = 33]	Help-Seeking/Attenuated Syndrome (stage 1) [N = 23]	Discrete Disorder/Persistent or Recurrent Illness (stage 2/3) [N = 24]	Significance Test (df) [p value]
Sex (f/m)	21/12	15/8	10/14	χ2 (80) = 3.5 [.171]
Age, years	23.9 ± 2.3	20.4 ± 5.2	23.5 ± 3.5	t (38.5) = -2.4 [.023]
Education, years	15.2 ± 2.2	12.1 ± 3.0	13.1 ± 2.6	t (38) = -1.1 [.281]
Predicted IQ	105.8 ± 8.5	105.0 ± 6.6	105.1 ± 6.6	t (39) = 0.1 [.899]
SOFAS	91.9 ± 3.5	65.3 ± 9.9	58.7 ± 13.4	t (34) = 1.7 [.104]
K-10 total	15.3 ± 6.1	26.4 ± 7.3	22.0 ± 7.0	t (40) = 2.0 [.055]
HDRS total	1.7 ± 1.7	10.6 ± 6.7	10.2 ± 7.6	t (36) = 0.2 [.878]
BPRS total	26.2 ± 2.2	37.1 ± 7.5	42.4 ± 10.7	t (35) = -1.7 [.090]

Mean scores (± standard deviation) for demographic and clinical variables across groups. Statistical tests are between stage 1 and stage 2/3 cohorts. Group differences were tested using chi-square or -test.

For symptoms potentially indicative of more severe syndromes, 35% of the help-seeking/attenuated syndrome group reported bipolar-like symptoms compared to 30% of the discrete disorder/persistent illness group; whereas only 9% of the help-seeking/attenuated syndrome group reported psychotic symptoms compared to 54% of the discrete disorder/persistent illness group. With regards to medication, less than 13% of cases were receiving no medication, and antidepressants were used equally across the different stages. However, more individuals in the later stages tended to receive additional antipsychotic (48% vs. 71%) or mood stabilizing (13% vs. 46%) medicines (see Table 2).

**Table 2:** Medication variables

Current Medication	Help-Seeking/Attenuated Syndrome [Count (%)]	Discrete Disorder/Persistent or Recurrent Illness [Count (%)]
Nil	3 (13.0)	2 (8.3)
Any Antidepressant	14 (60.8)	13 (54.2)
Any Antipsychotic	11 (47.8)	17 (70.8)
Any Mood Stabiliser	3 (13.0)	11 (45.8)

Cross-tabulation of clinical stage group by current medication.

### Imaging data

**Figure 1:**
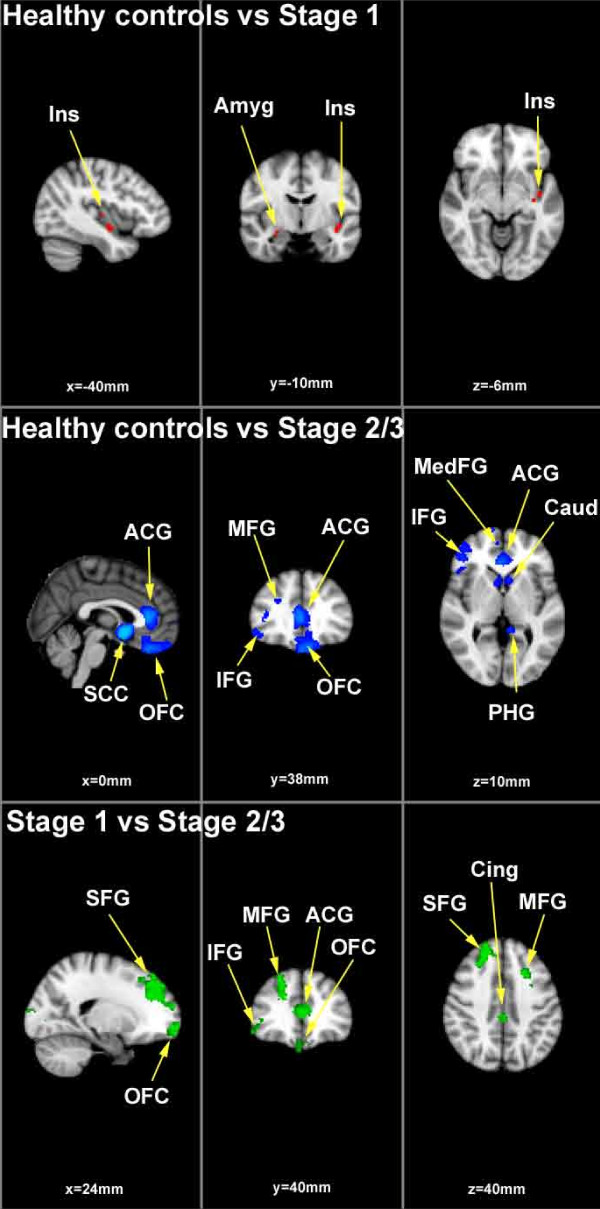
**VBM grey matter results**. Group difference, grey matter volume maps rendered on the MNI152 structural template. Statistically significant clusters of grey matter changes calculated using 23 patients at stage 1 (i.e. help-seeking/attenuated syndrome), 24 patients at stage 2/3 (i.e. discrete disorder/persistent or recurrent illness) and 33 healthy control subjects. Contrast 1 depicts healthy control subjects versus stage 1 subjects (grey matter loss occurring at stage 1 is presented in orange). Contrast 2 depicts healthy controls versus stage 2/3 (grey matter loss is presented in blue). Finally contrast 3 depicts grey matter volume changes in stage 1 versus stage 2/3 subjects (regions of significant grey matter loss are presented in green). Abbreviations are as follows: SFG = superior frontal gyrus; OFC = orbito frontal cortex; IFG = inferior frontal gyrus; MFG = middle frontal gyrus; ACG = anterior cingulate gyrus; Cing = cingulate; Ins = insula; Amyg = amygdala; MedFC = medial frontal gyrus; Caud = caudate; PHG = parahippocampal gyrus; SCC = subcallosal cortex. Images are radiologically oriented.

Healthy controls vs stage 1: Loss of grey matter volume was evident in the amygdala and precuneus on the right side as well as the insula on the left for the stage 1 subjects (see Table 3 and Figure 1).

**Table 3:** Grey matter results

Cortical Area	Hemisphere	MNI Coordinates	Brodmann Area	**Cluster size (mm**^**3**^**)**	Corrected p-value
Healthy Control vs Attenuated Sydnrome					
Amygdala	right	28, -8, -14		2894	0.05
Precuneus	right	14, -74, 34	7	3710	0.05
Insula	left	-42, -8, -8	13, 14	4156	0.05
					
Healthy Control vs Discrete Disorder					
Middle frontal gyrus	right	26, 46, 34	46	9088	0.01
Anterior cingulate cortex	midline	0, 36, 12	24	6748	0.01
Inferior frontal gyrus	right	46, 40, 10	10	4034	0.01
Caudate nucleus (head)	right	6, 12, 2		1799	0.01
Caudate nucleus (head)	left	-6, 12, 2		1907	0.01
Parahippocampal gyrus	left	-10, -42, 2	36	2019	0.01
Insula	left	-36, -24, 12	13, 14	5973	0.01
Medial prefrontal cortex	midline	0, 32, -16	9	9634	0.002
Orbitofrontal cortex	midline	0, 34, -26	10	9858	0.01
Subcallosal cortex	midline	0, 10, -8		7590	0.01
					
Attenuated Sydnrome vs Discrete Disorder					
Superior frontal gyrus	right	26, 30, 38	8, 9	8942	0.01
Middle frontal gyrus	right	36, 30, 24	46	7429	0.01
Middle frontal gyrus	left	-24, 22, 40	46	3830	0.01
Inferior frontal gyrus	right	40, 40, 8	10	6861	0.01
Inferior frontal gyrus	left	-46, 36, 8	10	4102	0.01
Anterior cingulate cortex	right	2, 40, 20	24	3642	0.02
Orbitofrontal cortex	right	26, 54, -4	10	4296	0.01
Medial prefrontal cortex	midline	0, 22, 20	9	3487	0.004

Results of significant clusters of grey matter volume loss for the right and left hemispheres for the following contrasts: (i) healthy controls vs attenuated syndrome, (ii) healthy controls vs discrete disorder and (iii) attenuated syndrome vs discrete disorder. Cortical coordinates are reported in MNI space, and cluster size in mm^3^

Healthy controls vs stage 2/3: Significant loss of grey matter volume was evident for the stage 2/3 group as compared to the controls in the following brain regions: middle and inferior frontal gyri on the right and medial prefrontal, orbitofrontal and subcallosal cortices, midline. Significant grey matter loss was also present in the left insula and parahippocampal gyrus as well as the head of caudate, bilaterally (see Table 3 and Figure 1).

Stage 1 vs stage 2/3: Significantly decreased grey matter volume was evident for the discrete disorder/persistent illness sample, only (see Table 3 and Figure 1). The largest cluster was located on an overlapping region between the superior and middle frontal gyrus on the right side. The greatest grey matter volume loss occurred predominantly in the superior frontal gyrus from which it extended inferio-anteriorly to the middle frontal gyrus. Grey matter volume was also significantly decreased in the middle frontal gyrus on the left, the inferior frontal gyrus and anterior cingulate on the right cerebrum and the medial prefrontal cortex midline and orbitofrontal cortex on the right.

## Discussion

This study investigated the extent to which grey matter loss occurs across clinical stages in young outpatients with an admixture of depression and other common psychiatric symptoms. Significant loss of frontal grey matter volume is characteristic of those in later stages where patients exhibit symptoms consistent with a discrete or persisting illness as compared with their peers who have earlier 'attenuated' syndromes. The observed pattern was characterised by a distributed loss of grey matter across a major portion of the prefrontal cortex with the greatest loss occurring within an overlapping region bounded by the superior and middle frontal gyri on the right side. Additional loss of grey matter volume was also observed in the inferior aspects of the frontal gyrus as well as the anterior cingulate and the orbitofrontal cortex on the right side as well as the medial prefrontal cortex midline. The pattern of grey matter loss also extended to the middle and inferior frontal gyri on the left side. When compared to healthy control subjects, stage 2/3 patients exhibited a similar pattern of grey matter loss, to that identified when compared to patients with attenuated symptoms, although the degree of volume loss was more extensive in the former comparison. In contrast when patients with attenuated symptoms were compared to controls the extent of the grey matter loss was markedly less. The results from this comparison indicated that the insula, amygdala and the precuneus were the initial brain regions affected. However, interestingly, grey matter changes within the amygdala and precuneus did not reach statistical significance for the more severe stage 2/3 group. This may suggest the grey matter loss observed in these regions for the stage 1 patient group might be related to the patient cohort per se rather than it being an early indicator of a disease trajectory. Longitudinal studies with larger patient cohorts would need to be conducted so as to explicate this finding.

The observed patterns of grey matter loss occurring across the clinical stages and healthy controls support our hypothesis that grey matter changes are more extensive in those with later or discrete disorders, as compared to those with earlier sub-syndromal or attenuated syndromes. Our results are consistent with those few other studies that have employed similar staging paradigms, that is, to categorise patients with psychotic disorders on the basis of illness progression rather than predefined diagnostic criteria [2,16]. While there is overlap between the patterns of grey matter loss observed in those studies (particularly within the prefrontal cortex) when compared to our current study, there are also important differences. Specifically, in these patients who present predominantly with depressive disorders, we did not find any evidence of grey matter loss that extended outside the prefrontal cortex. Those studies (largely of patients with emerging psychosis) that have employed a similar staging paradigm (both cross sectional and longitudinally) have reported more widespread grey matter loss that encompasses regions including the parahippocampal, fusiform and cerebellar cortices [2], temporal lobes [17] as well as the cerebellum and amygdala [18].

Whilst the changes in later stages were confined to the prefrontal cortex, they do bear similarities to those seen in 'high-risk' (or attenuated psychosis) patients before and after transitioning to first episode psychosis. Most of these studies have reported predominant right side pathology that incorporate the orbitofrontal, superior, medial cortices as well as the anterior cingulate cortex [16]. Indeed the established schizophrenia/psychosis literature is burgeoning with volumetric studies that report loss of grey matter in the aforementioned regions but invariably to a greater extent both in terms of spatial distribution but also degree of grey matter loss. Our patient cohort was comprised of help seeking adolescents and young adults who presented with a variety of risk criteria and attenuated anxiety, depressive, hypomanic and/or psychotic symptoms. As such, our patients constitute a very heterogeneous group, with a significant percentage likely to go on and develop more persistent affective or psychotic disorders. However, we expect that they will not all take the same trajectory and that only a small minority may ever transition to well-established major depressive, bipolar or psychotic disorders [19]. These differences in illness trajectories are likely to explain differences between our study and those, which are based on more narrowly, focused cohorts of patients selected to be at risk of psychosis. Interestingly, the few longitudinal studies that have been conducted on at-risk psychosis groups, have reported significantly decreased grey matter volume within the inferior frontal gyrus and superior temporal gyrus (both on the right side) in those patients that transitioned into full-blown disease [20]. This suggests that in the case of more pure forms of psychosis, the disease process preferentially targets these two regions.

In our current study we did not find any evidence of grey matter loss in the superior temporal gyrus however we did detect significant grey matter loss in the inferior frontal gyrus (bilaterally) amongst other prefrontal cortical regions. Our results would suggest that the inferior frontal gyrus may be a region that is targeted more broadly by a range of evolving psychiatric disorders and might not be confined only to the development of psychosis. If this finding can be replicated and is indeed robust, this could potentially provide a neuroimaging target that could be monitored in patients who may be, in the first instance help seeking or that carry an increased risk of developing a major psychiatric illness.

It is striking that our findings of decreased grey matter volume were predominantly confined to the prefrontal cortex and interestingly to specific regions therein which are putatively involved in the integration and regulation of behaviours. The superior and middle frontal gyri are regions widely reported to be involved in the executive functioning including decision making [21] and memory [22] both of which were found to be compromised in our more severe cohort. Moreover, the anterior cingulate (and more specifically its ventral aspect) and the orbitofrontal cortex are both involved with emotion-based decision making including the evaluation of risk and reward [23]. The structural changes uncovered within these areas are consistent with the wide-ranging symptoms exhibited by our patient cohort that includes mood, anxiety and psychotic symptoms.

There were several methodological limitations associated with our study. Firstly, the size for our patient cohorts, whilst adequately powered were modest in comparison to larger cross sectional studies that have investigated patients with established disease. As such we can't exclude the possibility that some grey matter changes (notably within some key temporal lobe structures) may not have attained statistical significance. A further limitation is associated with the potential confound of medication. Our discrete disorder/persistent illness cohorts were on average more likely to be receiving multiple medications than the help-seeking/attenuated syndrome group. Finally, longitudinal experimental designs with large cohorts are urgently needed (with scanning occurring prior to and following transition to a major psychiatric illness, and including patients who move to different eventual disorder outcomes such as bipolar and other discrete psychotic disorders) to separate out early shared changes from those that may be more characteristic of either later stages of all illnesses or isolated to particular clinical phenotypes.

## Conclusions

The results of our study indicate that there is a gradation of grey matter loss occurring in patients categorised by our clinical staging model. Patients presenting with a discrete or persistent illness (categorised as stage 2/3) had extensive grey matter loss predominantly within the prefrontal cortex as compared to help seeking individuals. Moreover, our findings suggest that discernable patterns of grey matter loss are indicative of stage of illness.

## Methods

Forty-seven health seeking participants aged 14 to 29 years were recruited from a specialised tertiary referral service for assessment and early intervention of mental health problems in young people [24,25] as part of a longitudinal study of youth mental health at the Brain and Mind Research Institute (BMRI), Sydney, Australia. Inclusion criterion for this sub-study were: i) persons aged 12-30 years seeking professional help primarily for significant anxiety, depressive, hypomanic or psychotic symptoms and, ii) willingness to participate in other neurobiological and longitudinal research with the BMRI related to clinical outcomes [26]. As such, this cohort represents a selected sub-set of a much broader cohort (n = 1483, 1260 of whom were aged 12-25 years) who presented to our services for clinical care during the same time period [25]. In addition 33 healthy control subjects (age range 15 to 28) were also recruited from the general population.

Subjects were excluded if they did not have sufficient English-language skills or had insufficient intellectual capacity to participate in the neuropsychological aspects of the concurrent studies [27]. The Human Research Ethics Committee of the University of Sydney approved this study, and all patients gave prospective written informed consent for their clinical data to be used for research purposes. Parental consent was obtained for patients under 18 years of age.

All patients who entered the services were assessed and managed by medically and/or psychologically-trained health professionals [28]. In this sub-study, an independent psychiatrist or trained research psychologist also conducted a standardized clinical interview, focusing on assessment of the detailed criteria developed for formal application of our clinical staging framework [13,28]. For those who are assessed in clinical environments, discrete categories (stages 1 to 4; whereby, stage 1a = 'help-seeking'; stage 1b = 'attenuated syndrome'; stage 2 = 'discrete disorder'; stage 3 = 'persistent or recurrent illness'; and stage 4 = 'chronic debilitating illness') are described. A key point of differentiation, however, occurs between those early 'sub-threshold' syndromes (classed as stage 1a or 1b) and the onset (stage 2) of a more discrete disorder. Importantly, entry to stage 2 was not simply analogous to, or defined by, meeting existing DSM or ICD criteria for a specific mood or psychotic disorder. At the time of scan 23 patients were identified to be at the 'attenuated syndrome' [stage 1b] (15 females) and 24 were rated to be within the 'discrete disorder' [stage 2] (N = 13; 5 females) or 'persistent or recurrent illness' [stage 3] (N = 11; 5 females) stage. The stage 2/3 cohorts included patients with a diagnosis of bipolar disorder, depression and psychosis. To ensure consistent inter-rater reliability estimates, two approaches were applied: i) first, we compared the determination of clinical stage by the original treating clinician with that assigned by the consensus pairs working from the records. The key comparison was for the degree of concordance between ratings of 'sub-syndromal or attenuated states' (stage 1) versus later established disorders (stages 2-4); ii) second, the ratings derived from a consensus pair were compared with that derived by an independent rater from our research team.

All imaging was conducted at the BMRI imaging facility using a 3T GE Discovery MR750 scanner (GE Medical Systems, Milwaukee, WI). Structural images were acquired using an 8-channel phased array head coil using a T1-weighted magnetization prepared rapid gradient-echo (MPRAGE) sequence producing 196 sagittal slices (TR = 7.2 ms; TE = 2.8 ms; flip angle = 10°; matrix 256 × 256; 0.9 mm isotropic voxels). For each subject, two T1-weighted MRI scans were obtained in single scan sessions, and individual structural whole brain MRI's were averaged to increase signal-to-noise ratio.

An unbiased optimised VBM analysis was carried out using FSL (FMRIB Software Library, http://www.fmrib.ox.ac.uk/fsl, Smith et al., 2004) with no a priori information regarding possible loci of structural changes in the grey matter. The FSL-VBM analysis pipeline was then conducted as follows. Firstly, brain tissue was extracted prior to tissue-type segmentation using BET [29]. All T1-weighted images were then transformed into standard space using a limited degrees-of-freedom non-linear model to ensure maximum spatial alignment and images were corrected for non-uniformity. The FAST4 [30] tool was then applied to segment tissues according to their type. The segmented grey matter partial volume images were then aligned into the MNI standard space by applying the affine registration tool FLIRT [31,32] and nonlinear registration FNIRT [33,34] methods, which use a B-spline representation of the registration warp field [35]. An averaged study-specific template was then created using all the unsmoothed images to which the grey matter partial volume images were re-registered and these registered images were then modulated to correct for local expansion and contraction by the Jacobian of the warp field. Rigorous visual inspection was used to ensure the quality of brain image extraction, segmentation, and registration for each averaged structural image. The modulated, segmented images were then smoothed with an isotropic Gaussian kernal with 3 mm standard deviation (FWHM 7.05 mm). The most common quality control issue encountered was suboptimal brain extraction using the BET tool. This was evident in brain-extracted images, which had unwanted skull and neck tissue included as part of the final processed image. In such instances it was necessary to re-run the brain extraction step using the "bias field correction and neck cleanup" option in BET.

Finally, permutation-based non-parametric testing, using a 5000 permutation set was used in a voxel-wise GLM [36] for contrasting differences between (i) healthy controls versus stage 1 patients (help-seeking/attenuated syndrome), (ii) healthy controls versus stage 2/3 (discrete disorder/persistent or recurrent illness) which depicts a more established disease process and finally (iii) stage 1 versus stages 2/3. To identify regionally specific associations that were not confounded by differences in age and total intracranial volume across the three groups, both these variables were entered into the design matrix as confounding covariates. Family-wise error (FWE) correction [37] was used to correct the threshold for multiple comparisons across space and threshold-free cluster enhancement was employed to assess cluster significance [38]. The significant p-value with the FWE corrected threshold was set at p < 0.05.

## Competing interests

The authors declare that they have no competing interests.

## Authors' contributions

JL carried out the imaging analysis, and drafted the manuscript. IBH, SLN and DFH conceived of the study, and participated in its design and coordination and helped to draft the manuscript. EMS participated in the design of the study and performed the clinical assessments. All authors contributed significantly to the interpretation of the data as well as having read and approved the final manuscript.

## Pre-publication history

The pre-publication history for this paper can be accessed here:

http://www.biomedcentral.com/1471-244X/12/4/prepub
